# Association between measurements of arterial stiffness and target organ damage in a general Spanish population

**DOI:** 10.1080/07853890.2021.1881812

**Published:** 2021-02-03

**Authors:** Rosario Alonso-Domínguez, Natalia Sánchez-Aguadero, María C. Patino-Alonso, Cristina Agudo-Conde, Ángela de Cabo-Laso, Marta Gómez-Sánchez, Leticia Gómez-Sánchez, Emiliano Rodríguez-Sánchez, Luis García-Ortiz, Manuel A. Gómez-Marcos, On Behalf of the EVA Investigators

**Affiliations:** a Biomedical Research Institute of Salamanca (IBSAL), Primary Care Research Unit of Salamanca (APISAL), Health Service of Castile and Leon (SACyL), Salamanca, Spain; b Department of Nursing and Physiotherapy, University of Salamanca, Salamanca, Spain; c Department of Statistics, University of Salamanca, Salamanca, Spain; d Iberian Network on Arterial Structure, Central Hemodynamics and Neurocognition, Portugal and Spain; e Department of Medicine, University of Salamanca, Salamanca, Spain; f Department of Biomedical and Diagnostic Sciences, University of Salamanca, Salamanca, Spain

**Keywords:** Vascular stiffness, vascular structure, vascular function, target organ damage

## Abstract

**Introduction:**

Little is known about the relationship between arterial stiffness and cardiovascular target organ damage (TOD) in the general population. The aim was to analyse the relationship between different measurements of arterial stiffness and TOD, in a general Spanish population without a history of cardiovascular event.

**Materials and methods:**

Transversal descriptive study. Through stratified random sampling, a total of 501 individuals were included. Carotid-femoral pulse wave velocity (cf-PWV) was measured using a SphygmoCor System^®^, the cardio-ankle vascular index (CAVI) was determined with aVasera VS-1500^®^ and brachial-ankle pulse wave velocity (ba-PWV)was calculated through a validated equation.

**Results:**

The average age was 55.84 ± 14.26.The percentage of vascular TOD, left ventricular hypertrophy (LVH) and renal TOD was higher in men (*p* < .001). A positive correlation was obtained between carotid intima-media thickness (c-IMT) and the measurements of vascular function. In the model 1 of the logistic regression analysis, cf-PWV was associated with vascular TOD (OR = 1.15, *p* = .040), ba-PWV was associated with vascular TOD (OR = 1.20, *p* = .010) and LVH (OR = 1.12, *p* = .047).

**Conclusions:**

The different measurements of arterial stiffness are highly associated with each other. Moreover, cf-PWV and ba-PWV were associated with vascular TOD, and ba-PWV with LVH, although they disappear when adjusting for cardiovascular risk factors.

**Key Messages**
- There is a strong correlation between the different measurements of vascular structure and function.
- Carotid-femoral and brachial-ankle pulse wave velocity were positively associated with vascular target organ damage, the latter was also positively associated with left ventricular hypertrophy.
- This associations disappear when adjusting for cardiovascular risk factors.

## Introduction

Arterial stiffness is an independent predictor of cardiovascular morbimortality [1–3], and there are numerous indices to assess it [4]. Carotid-femoral pulse wave velocity (cf-PWV) is considered the gold standard in the western population [5]. However, brachial-ankle pulse wave velocity (ba-PWV) is the most used method in eastern countries [6–8]. The main difference between both is the type of vessel included in their measurements, since ba-PWV includes a greater number of non-aortic vessels, as well as a large part of medium sized and resistive arteries [9]. However, several studies [4,10,11] show that there is a strong correlation between these two measurements and they have both been associated in the clinical practice with cardiovascular risk [12]. Nevertheless, they have some limitations: the technique to measure cf-PWVrequires previous training and has a lower reproducibility thanba-PWV [13], and both measurements depend on the level of blood pressure recorded at the same time [14]. On the other hand, the cardio-ankle vascular index (CAVI) [15,16] does not have these drawbacks, as it does not depend on the level of blood pressure at the time it is measured, and it is easy to use in the clinical practice. This index has been previously associated with cf-PWV and ba-PWV in the study conducted by Gómez-Marcos et al. [17] in diabetic patients. However, in the general population, Alieva et al. [18] found that the different markers of arterial stiffness had weak correlations among them and, thus, could not be interpreted as interchangeable measurements of arterial stiffness.

Another factor to take into account in the monitoring of cardiovascular risk in the general population is target organ damage (TOD). This damage is considered the intermediate point that links cardiovascular risk factors and cardiovascular events to mortality [19]. Thus, the European guidelines for the management of arterial hypertension [7] recommend its use in the assessment of cardiovascular risk, since, in general, TOD tends to be asymptomatic [20] and patients usually present it in combination with other symptoms [1]. Currently, there are discrepancies among the different studies that have evaluated the relationship between the measurements of arterial stiffness and the presence of TOD. Thus, CAVI has been associated with carotid intima-media thickness (c-IMT) and with the presence of carotid plaque in the general population [13] and in diabetic patients [17]. In patients with arterial hypertension, cf-PWV and ba-PWV have been associated with c-IMT and microalbuminuria [19,21,22], and CAVI has been associated with c-IMT [23] and left ventricular hypertrophy (LVH) [24].

Therefore, the aim of this study was to analyse the relationships among measurements of arterial stiffness and their association with TOD, defined according to criteria established in the 2018 European hypertension guideline, in a general Spanish population without a history of cardiovascular disease.

## Materials and methods

### Study design

“Influence of Different Risk Factors in Vascular Accelerated Aging” (EVA Study) (NCT02623894) [25] is a cross-sectional study.

### Characteristics and participants

The study was carried out in the scope of primary care in Salamanca, Spain. The participants were selected through stratified random sampling based on age and sex (35, 45, 55, 65 and 75 years). A total of 501 participants were included, with 100 participants in each age group and a 1:1 sex ratio for all groups. The recruitment was conducted from June 2016 to November 2017. A detailed description of the methodology, the inclusion and exclusion criteria and the response rate was previously published [25]. The flow chart that describes the reference population (43,946), the included and excluded individuals and the causes of exclusion are shown in Supplementary Figure 1S of the Supplementary material.

### Ethical considerations

The study was approved by the Drug Research Ethics Committee of the Health Area of Salamanca on May 4^th^ 2015. All procedures performed in the study involving human participants were in accordance with the ethical standards of the institutional and/or national research committee and with the 2013 Declaration of Helsinki [26]. All participants signed written informed consent documents prior to participation in the study.

### Variables and measurement instruments

The researchers involved in the collection of biological samples, the realisation of physical examinations and the gathering of the data analysed in the EVA study were previously trained following a standardised protocol.

#### Vascular function and structure

##### Carotid-femoral pulse wave velocity

Carotid-femoral Pulse Wave Velocity was measured using a SphygmoCor^®^ device (At Cor Medical Pty Ltd Head Office, West Ryde, Australia), with the patient in the supine position. This parameterwas calculated by estimating the delay of the pulse wave with respect to the wave of the electrocardiogram (ECG). The distance was measured from the sternal notch to the point where the sensor was placed (carotid and femoral artery). Any value of cf-PWV>10 m/s was considered pathological [27]. The pulse wave was measured with a sensor in the radial artery, using mathematical transformations to estimate the aortic pulse wave.

##### Cardio-Ankle vascular index, brachial-ankle pulse wave velocity and Ankle-Brachial index

These parameter were measured using a Vasera VS-1500^®^ device (FukudaDenshi), after introducing the information of each participant and following the instructions of the manufacturer. The measuring was conducted in a room at constant temperature. The participants were requested to fast for the test, to avoid smoking or drinking coffee one hour before the measuring, to wear comfortable clothes and to be at rest for 10 min before the test. The CAVI values were calculated automatically by replacing the β-stiffness index in the following equation: β-stiffness index = 2ρ x 1/(Ps – Pd) × In (Ps/Pd) ×PWV^2^, where ρis blood density, and PS and PD are systolic blood pressure (SBP) and diastolic blood pressure (DBP),respectively, in mmHg [28]. Any level of CAVI≥ 9 was considered abnormal, with suspected subclinical atherosclerosis. Brachial-ankle Pulse Wave Velocity was estimated using the following equation: ba-PWV= (0.5934 × height (cm) +14.4724)/TBA, where TBA is the time interval between the arm and ankle waves [29]. Any value of ba-PWV ≥17.5 m/s was considered abnormal [30]. The ankle-brachial index (ABI)was calculated automatically for each foot dividing foot SBP by arm SBP. Any ABI value below 0.9 or above 1.3 was considered pathological [31].

##### Carotid Intima-Media Thickness

Carotid intima-media thickness was assessed using a Sonosite Micromaxx^®^ ultrasound scanner (Sonosite Inc., Bothell, Washington, USA), coupled to a high-definition linear transducer at a multifrequency of 5-10 MHz, along with the Sonocal software to measure this parameter automatically, with the aim of optimising the reproducibility of the assessment. The c-IMT values were measured with the participant lying down, with his/her head extended and slightly turned in the opposite direction to the examined artery. The common carotid artery was measured after examining a longitudinal section of 10 mm at 1 cm from the bifurcation, recording the thickness of the proximal and distal wall in the lateral, anterior and posterior projections. Values of c-IMT>0.9 mm and the presence of a plaque were considered pathological, considering the latter when c-IMT ≥1.5 mm or when there was a focal increase of either 0.5 mm or 50% of the c-IMT of the adjacent carotid, following the2018 ESC/ESH guidelines for the diagnosis and treatment of arterial hypertension [32].

#### Cardiovascular risk factors

Clinical arterial pressure was measured using a validated OMRON M10-IT^®^ sphygmomanometer (Omron Health Care, Kyoto, Japan), following the recommendations of the European Society of Hypertension [33].

Body weight was determined with the participant barefoot and light clothes, recording the mean of two measurements, using an authorized Seca 770^®^electronic scale (Medical scale and measurement system, Birmingham, UK), which was previously calibrated. Height was measured with the participant in standing position and barefoot, using a Seca 222^®^height rod (Medical scale and measurement system, Birmingham, UK), recording the mean of two measurements. The body mass index (BMI) was calculated dividing body weight (kg) by height in metres squared (m^2^).

Participants were considered to have hypertension if they took antihypertensive drugs or had arterial pressure values ≥140/90 mmHg. Participants were considered diabetic if they took hypoglycaemic drugs or had blood glucose levels ≥126 mg/dl or values of glycosylated haemoglobin (HbA1c) ≥6.5%. Participants were considered to have dyslipidemia if they took hypolipidemic drugs or had fasting levels of total cholesterol≥240 mg/dl, or low-density lipoprotein-bound cholesterol (LDL-c) ≥160 mg/dl, or high-density lipoprotein-bound cholesterol (HDL-c) ≤40 mg/dl in menand ≤50 mg/dl in women, or triglycerides ≥200 mg/dl. Participants with a BMI ≥30 were considered obese. Participants were considered to be smokers if they smoked at the time of the test or had stopped smoking less than one year before the test.

Blood samples taken between 8 and 9 a.m. after at least 12 h of fasting were used to determine the plasma glycaemia, HbA1c, total cholesterol, HDL-cand triglycerides, employing standard automatizedenzymatic methods. LDL-c was calculated using Friedewald’sformula [34].

#### Target organ damage

To define the TOD criteria, the 2018 ESC/ESH Guidelines for the diagnosis and treatment of arterial hypertension were followed [32]. Vascular TOD was considered in patients who showed ABI <0.9, IMT-*c* ≥ 0.9 mm or a carotid plaque in the ultrasound scan. LVH was considered if the Sokolow–Lyon index was above 35 mm or the Cornell voltage duration product was above 2440 mm*ms.Renal TOD was considered in patients with microalbuminuria (30–300 mg/24 h), elevated albumin–creatinine ratio (30–300 mg/g) or moderate chronic kidney disease with eGFR 30–59ml/min/1.73m^2^ estimated with the CKD-EPI equation [32].

#### Evaluation of lifestyles

The smoking status was collected with a standardised questionnaire (recording whether the participant was a smoker or not, and the frequency of the habit). In smokers and former smokers, the number of years they had been smokers was also recorded. Alcohol consumption was also collected with a standardised questionnaire (recording the type and amount of alcohol ingested during a week, measuring it in gr/week). Adherence to the Mediterranean diet was evaluated with a 14-item questionnaire validated in Spain and used in the PREDIMED study. Scores ≥9 were considered “good adherence” [35]. Physical activity was assessed objectively using an accelerometer (*Actigraph^®^*, Shalimar, FL, USA), which had been previously validated [36]. Subjects wore the accelerometer attached to the right side of the waist for seven consecutive days, except for bathing and water activities. The data were recorded at 1-minute intervals. Total physical activity was expressed in hours/week. The accelerometer gives us data on the intensity of the activity carried out by the subject and sedentary time in hours/day. The variable analysed in this study was total physical activity (hours/week).

### Statistical methods

The data of the continuous variables are presented as mean ± standard deviation and the categorical variables are expressed in numbers and percentages. The measurements were compared by sex using Student’s t-test or the Chi-square test, depending on the type of variable. The correlation between the different measurements of arterial stiffness and the cardiovascular risk factors and parameters that determine vascular and renal TOD, and LVH was analysed using Pearson’s correlation test. Three multiple linear regression analyses were conducted, consideringc-IMT, ABI, Sokolow–Lyon index, PDV-Cornell, albumin creatinine ratio and the glomerular filtration rate estimated with the Chronic Kidney Disease Epidemiology Collaboration equation (CKD-EPI) as dependent variables and cf-PWV, ba-PWV and CAVI as independent variables. Three logistic regression analyses were also conducted, considering the presence of vascular and renal TOD, and LVH as dependent variables (1= presence of TOD, 0= absence of TOD) andcf-PWV, ba-PWV and CAVI as independent variables. In both the multiple and logistic regressions, we used age, sex, years of smoking, grams of alcohol ingested per day, physical activity and adherence to a Mediterranean diet as adjusted variables in the first model of adjustment. In the second model of adjustment, we used these variables as well as SBP, BMI, fasting blood glucose and triglycerides.

A Receiver Operating Characteristic analysis (ROC) was conducted to determine the capacity of the different measurements of arterial stiffness to diagnose vascular and renal TOD, and LVH. The area under the ROC curve (AUROC) and the 95% confidence intervals (CI) were calculated with the aim of comparing the discriminatory power of each measurement of arterial stiffness.

The data were analysed using SPSS Statistics for Windows v.25.0 (IBM Corp, Armonk, NY). For the bilateral hypothesis tests, the alpha risk was set at 0.05 as the limit of statistical significance.

## Results

### Participants

The characteristics of the participants in general and by sex are presented in Table 1, including the lifestyles, conventional cardiovascular risk factors, the presence of vascular and renal TOD, LVH, and the drugs used in the treatment of hypertension, diabetes mellitus and dyslipidemia. The mean age was 55.84 ± 14.26 years. With respect to the women, the men consumed more alcohol, practiced less physical activity and had a lower adherence to the Mediterranean diet. They also showed higher values in arterial pressure, BMI, glycaemia and triglycerides, and lower levels of HDL-c. Furthermore, the men had greater percentages of vascular TOD (17.1% vs 7.5%), LVH (24.4% vs 6.0%) and renal TOD (16.7% vs 7.2%), and higher values of cf-PWV and CAVI.

**Table 1.** Baseline demographic and clinical characteristics of participants by sex.

	Overall (*n* = 494)	Men (*n* = 245)	Women (*n* = 249)	*p* Value
Lifestyles				
Alcohol, (gr/W)	46.18 ± 78.49	72.00 ± 95.90	20.78 ± 43.49	<.001
Smoker, *n* (%)	88 (17.80)	49 (19.70)	41 (16.30)	.430
Smoker, (years)	12.77 ± 17.27	14.01 ± 18.68	11.56 ± 15.70	.115
Mediterraneandiet	7.15 ± 2.08	6.69 ± 1.97	7.61 ± 2.09	<.001
Total physical activity, (h/W)	8.09 ± 0.66	7.86 ± 0.50	8.30 ± 0.73	<.001
Cardiovascular risk factors				
Age, years	55.84 ± 14.26	55.87 ± 14.34	55.81 ± 14.20	.962
Family history of premature CVD, *n* (%)	199 (40.3)	96 (38.6)	107 (42.5)	.389
BMI, kg/m^2^	26.41 ± 4.08	26.85 ± 3.49	25.97 ± 4.55	.017
SBP, mmHg	120.52 ± 23.11	126.29 ± 19.40	114.85 ± 25.03	<.001
DBP, mmHg	75.42 ± 10.10	77.29 ± 9.36	73.58 ± 10.48	<.001
FPG, mg/dl	88.08 ± 17.23	89.82 ± 18.44	86.37 ± 15.79	.026
HbA1c, %	5.49 ± 0.55	5.53 ± 0.61	5.44 ± 0.47	.070
T-c, mg/dl	194.56 ± 32.49	192.11 ± 32.11	196.98 ± 32.75	.096
LDL-c, mg/dl	115.28 ± 29.32	117.03 ± 29.95	113.57 ± 28.64	.192
HDL-c, mg/dl	58.83 ± 16.13	53.19 ± 13.95	64.34 ± 16.24	<.001
Triglycerides, mg/dl	102.80 ± 53.34	111.73 ± 54.56	94.02 ± 50.71	<.001
Target organ damage				
c-IMT, mm	0.68 ± 0.11	0.70 ± 0.12	0.66 ± 0.10	.001
Vascular TOD, *n* (%)	58 (11.7)	39 (17.1)	19 (7.5)	.004
ABI	1.11 ± 0.08	1.13 ± 0.08	1.10 ± 0.08	<.001
Cornell	13.22 ± 5.85	14.64 ± 6.14	11.81 ± 5.20	<.001
VDP Cornell	1479.27 ± 607.68	1423.11 ± 711.42	1534.98 ± 478.33	.041
Sokolow	20.67 ± 6.61	22.17 ± 6.85	19.18 ± 6.01	<.001
LVH, *n* (%)	74 (15.0)	60 (24.4)	15 (6.0)	<.001
Creatinine, mg/dl	0.80 ± 0.19	0.91 ± 0.17	0.69 ± 0.13	<.001
CKD-EPI, mL/(min.1.73 m^2^)	93.26 ± 16.29	91.66 ± 16.18	94.83 ± 16.27	.030
ACR, mg/g	14.13 ± 39.26	10.86 ± 31.54	17.36 ± 45.46	.067
Renal TOD, *n* (%)	59(11.9)	41 (16.7)	18 (7.2)	.001
Diseases and treatment				
Hypertension, *n* (%)	143 (28.9)	82 (32.9)	65 (25.8)	.109
Dyslipidemia, *n* (%)	320 (64.8)	162 (65.6)	164 (65.1)	.993
Diabetes mellitus, *n* (%)	37 (7.5)	26 (10.4)	12 (4.8)	.023
Obesity, *n* (%)	89 (18.0)	42 (16.9)	52 (20.6)	.332
Antihypertensive treatment, *n* (%)	92 (18.6)	50 (20.1)	46 (18.3)	.751
Lipid-lowering treatment, *n* (%)	102 (20.6)	49 (19.7)	53 (21.0)	.724
Antidiabetic treatment, *n* (%)	34 (6.9)	23 (9.2)	12 (4.8)	.068
Vascular function				
cf-PWV, m/s	8.15 ± 2.49	8.54 ± 2.68	7.75 ± 2.23	<.001
ba-PWV, m/s	12.92 ± 2.67	13.14 ± 2.45	12.71 ± 2.86	.070
CAVI	8.01 ± 1.44	8.13 ± 1.49	7.88 ± 1.39	.049

The continuous variables were represented as average ± standard deviation; the categorical variables as number and percentage.

^a^*p* Value show differences between men and women.

W: week; CVD: cardiovascular disease; BMI: body mass index; SBP: systolic blood pressure; DBP: diastolic blood pressure; FPG: fasting plasma glucose; HbA1c: glycosylated haemoglobin; T-c: total cholesterol; LDL-c: low density lipoprotein cholesterol; HDL-c: high density lipoprotein cholesterol; c-IMT: carotid intima-medium thickness; ABI: ankle-brachial index; TOD: target organ damage; LVH: left ventricular hypertrophy; VDP: voltage-duration product; CKD-EPI: chronic kidney disease epidemiology collaboration; ACR: albumin-creatinine ratio; cf-PWV: carotid-femoral pulse wave velocity; ba-PWV: brachial-ankle pulse wave velocity; CAVI: cardio-ankle vascular index.

**Figure 1.**
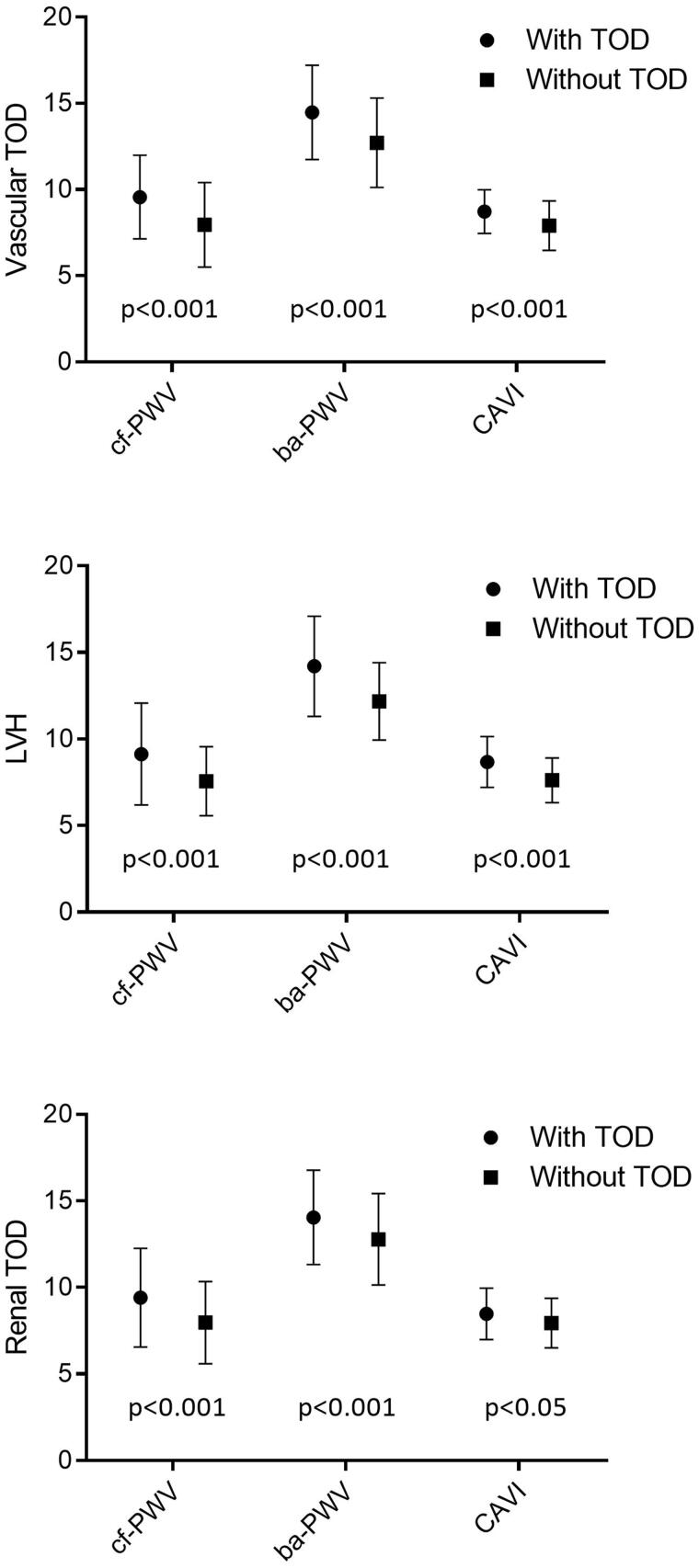
Estimated average and standard deviation of cf-PWV, ba-PWV and CAVI in subjects with and without vascular and renal TOD, and LVH. *p*-value: differences among groups. TOD: target organ damage; LVH: left ventricular hypertrophy; cf-PWV: carotid-femoral pulse wave velocity; ba-PWV: brachial-ankle pulse wave velocity; CAVI: cardio-ankle vascular index.

**Figure 2.**
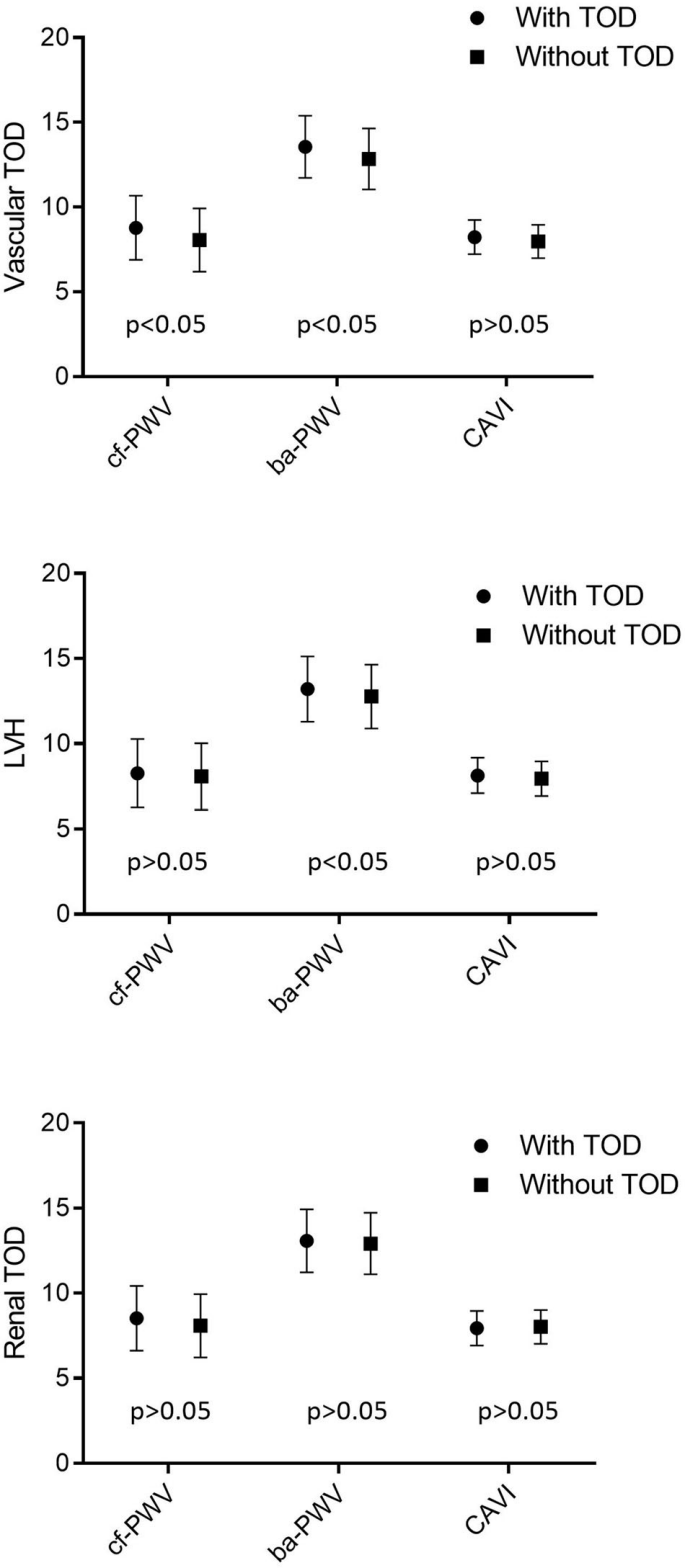
Estimated average and standard deviation of cf-PWV, ba-PWV and CAVI in subjects with and without vascular and renal TOD, and LVH. Adjusted for age (years) and sex (0= female, 1 = male). *p*-value: differences among groups. TOD: target organ damage; LVH: left ventricular hypertrophy; cf-PWV: carotid-femoral pulse wave velocity; ba-PWV: brachial-ankle pulse wave velocity; CAVI: cardio-ankle vascular index.

The mean values of cf-PWV, ba-PWV and CAVI in the participants with vascular and renal TOD, and LVH were higher than those in participants who did not have any type of TOD (*p* < .05 in all cases) (Figure 1). After adjusting by age and sex (Figure 2), the differences of the three measurements were only maintained between participants with vascular TOD and those without vascular TOD measured through cf-PWV and ba-PWV (*p* < .05), and between participants with LVH and those without LVH measured through ba-PWV (*p* < .05).

### Correlation of cf-PWV, ba-PWV and CAVI with cardiovascular risk factors and TOD

The correlations between the variables of vascular structure and function, and between these and cardiovascular risk factors are presented in Table 2. The values of c-IMT and the measurements of vascular function showed a positive correlation, with r values between 0.095 (*p* < .05) (ABI and c-IMT) and 0.831 (*p* < .01) (CAVI and ba-PWV).

**Table 2.** Bivariate correlations of structure and function parameters with cardiovascular risk factors.

	c-IMT	cf-PWV	ba-PWV	CAVI
c-IMT	1	0.563**	0.623**	0.591**
ABI	0.095*	0.015	0.058	0.166**
cf-PWV	0.563**	1	0.734**	0.621**
ba-PWV	0.623**	0.734**	1	0.831**
CAVI	0.591**	0.621**	0.831**	1
Age	0.744**	0.641**	0.734**	0.727**
BMI, kg/m^2^	0.310**	0.249**	0.195**	0.026
SBP, mmHg	0.370**	0.339**	0.435**	0.301**
DBP, mmHg	0.219**	0.340**	0.446**	0.284**
FPG, mg/dl	0.284**	0.359**	0.313**	0.238**
HbA1c, %	0.353**	0.423**	0.375**	0.344**
T-c, mg/dl	0.169**	0.076	0.155**	0.115*
LDL-c, mg/dl	0.159**	0.045	0.107*	0.086
HDL-c, mg/dl	−0.047	−0.075	−0.021	0.017
Triglycerides, mg/dl	0.189**	0.229**	0.245**	0.127**
Sokolow	−0.101*	−0.050	−0.045	−0.072
VDP-Cornell	0.036	0.035	0.052	0.018
Creatinine, mg/dl	0.149**	0.166**	0.039	0.066
ACR, mg/g	0.041	0.020	0.110*	0.050
CKD-EPI, mL/(min.1.73 m^2^)	−0.503**	−0.439**	−0.418**	−0.434**

^a^*p*-value s were determined by Pearson correlation. *p < .05; **p < .01.

c-IMT: carotid intima-medium thickness; cf-PWV: carotid-femoral pulse wave velocity; ba-PWV: brachial-ankle pulse wave velocity; CAVI: cardio-ankle vascular index; ABI: ankle-brachial index; BMI: body mass index; SBP: systolic blood pressure; DBP: diastolic blood pressure; FPG: fasting plasma glucose; HbA1c: glycosylated haemoglobin; T-c: total cholesterol; LDL-c: low density lipoprotein cholesterol; HDL-c: high density lipoprotein cholesterol; VDP: voltage duration product; ACR: albumin-creatinine ratio; CKD-EPI: chronic kidney disease epidemiology collaboration.

### Association of cf-PWV, ba-PWV and CAVI with TOD

In model 1 of the multiple regression analysis, cf-PWV showed a positive association with c-IMT (β = 0.004; CI 95%: 0.001–0.008; *p* = .022) and a negative one with ABI (β = −0.005; CI 95%: −0.009 – −0.002; *p* = .006), ba-PWV was positively associated with c-IMT (β = 0.005; CI 95%: 0.002–0.009; *p* = .004), with the Sokolow-Lyon index (β = 0.374; CI 95%: 0.057–0.691; *p* = .021) and with the glomerular filtration rate estimated with the CKD-EPI equation (β = 0.997; CI95%: 0.394–1.601; *p* = .001), and CAVI showed a positive association with ABI (β = 0.009; CI 95%: 0.002–0.016; *p* = .015) and with CKD-EPI (β = 1.159; CI 95%: 0.044–2.275; *p* = .042). In the second multiple regression model, cf-PWV was negatively associated with ABI (β = −0.004; CI 95%: −0.008–0.000; *p* = .044) and with ACR (β = −2.221; CI 95%: −4.260–−0.182; *p* = .033). ba-PWV had a positive association with c-IMT (β = 0.006; CI 95%: 0.001–0.010; *p* = .007) and CKD-EPI (β = 1.316; CI 95%: 0.663–1.969; *p* < .001), and CAVI showed a positive association with c-IMT (β = 0.009; CI 95%: 0.003–0.016; *p* = .007) and with ABI (β = 0.010; CI 95%: 0.002–0.017; *p* = .009) (Table 3).

**Table 3.** Regression analysis of vascular structure and function parameters with vascular, cardiac, and renal parameters.

	Cf-PWV			ba-PWV			CAVI		
	β	(95% CI)	*p* Value	β	(95% CI)	*p* Value	β	(95% CI)	*p* Value
Vascular TOD									
c-IMT									
M1	0.004	0.001; 0.008	.022	0.005	0.002; 0.009	.004	0.005	− 0.001; 0.012	.108
M2	0.003	− 0.001; 0.006	.172	0.006	0.001; 0.010	.007	0.009	0.003; 0.016	.007
ABI									
M1	− 0.005	− 0.009; − 0.002	.006	− 0.002	− 0.006; 0.001	.225	0.009	0.002; 0.016	.015
M2	− 0.004	− 0.008; 0.000	.044	− 0.001	− 0.005; 0.003	.604	0.010	0.002; 0.017	.009
LVH									
Sokolow									
M1	0.114	− 0.189; 0.416	.460	0.374	0.057; 0.691	.021	0.271	− 0.313; 0.855	.362
M2	0.012	− 0.306; 0.331	.940	0.222	− 0.120; 0.563	.202	− 0.016	− 0.619; 0.586	.957
PDV − Cornell									
M1	14.096	− 13.934; 42.126	.324	13.293	− 17.158; 43.745	.391	− 16.042	− 71.974; 39.890	.573
M2	12.604	− 17.238; 42.446	.407	6.007	− 27.189; 39.204	.722	− 28.480	− 86.996; 30.037	.339
Renal TOD									
CKD-EPI									
M1	− 0.102	− 0.682; 0.479	.731	0.997	0.394; 1.601	.001	1.159	0.044; 2.275	.042
M2	0.071	− 0.546; 0.688	.821	1.316	0.663; 1.969	<.001	1.010	− 0.158; 2.178	.090
ACR									
M1	− 1.492	− 3.398; 0.414	.125	0.673	− 1.310; 2.656	.505	− 2.078	− 5.708; 1.552	.261
M2	− 2.221	− 4.260; − 0.182	.033	0.040	− 2.130; 2.210	.971	− 2.594	− 6.409; 1.222	.182

Model 1: Adjusted by age (years), smoking (years), sex, alcohol (gr), total physical activity (hour/week) and Mediterranean Diet (points).

Model 2: Adjusted by age (years), smoking (years), sex, alcohol (gr), total physical activity (hour/week) and Mediterranean Diet (points).

SBP (mmHg), BMI (kg/m^2)^, FPG (mg/dl), and Triglycerides (mg/dl).

^a^*p*-value for statistically significant differences (*p* < .05).

TOD: target organ damage; c-IMT: carotid intima-medium thickness; LVH: left ventricular hypertrophy; CKD-EPI: chronic kidney disease epidemiology collaboration; ACR: albumin creatinine ratio; β: correlation coefficient; CI: confidence interval;cf-PWV: carotid-femoral pulse wave velocity; ba-PWV: brachial-ankle pulse wave velocity; CAVI: cardio-ankle vascular index; ABI: ankle-brachial index.

**Figure 3.**
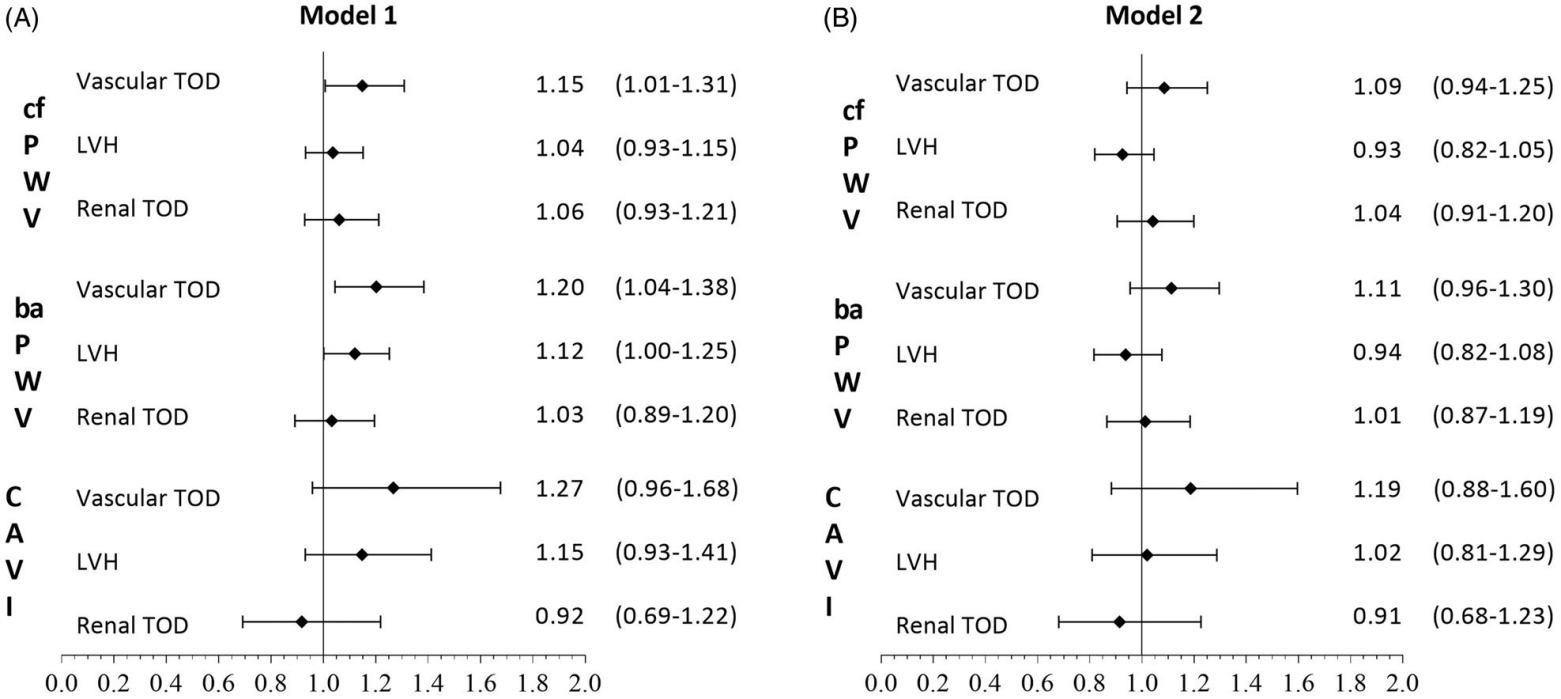
**A**. Logistic regression analysis adjusted by age (years), smoking (years), sex, alcohol (gr), total physical activity (hour/week) and Mediterranean Diet (points), OR of cf-PWV, ba-PWV and CAVI with vascular and renal TOD, and LVH. B. Logistic regression analysis adjusted by age (years), smoking (years), sex, alcohol (gr), total physical activity (hour/week), Mediterranean Diet (points), SBP (mmHg), BMI (kg/m^2^), FPG (mg/dl) and triglycerides (mg/dl), OR of cf-PWV, ba-PWV and CAVI with vascular and renal TOD, and LVH. TOD: target organ damage; LVH: left ventricular hypertrophy;cf-PWV: carotid-femoral pulse wave velocity; ba-PWV: brachial-ankle pulse wave velocity; CAVI: cardio-ankle vascular index.

In the first adjustment model of the logistic regression analysis (Figure 3), cf-PWV showed a positive association with vascular TOD (OR = 1.15; CI 95%: 1.01–1.31; *p* = .040). ba-PWV was positively associated with the presence of vascular TOD (OR = 1.20;CI 95%: 1.04–1.38; *p* = .010) and LVH (OR = 1.12; CI 95%: 1.00–1.25; *p* = .047), showing no significant association with renal TOD. CAVI was not associated with any of the assessed TOD types. The second logistic regression adjustment model yielded no significant associations.

### ROC curves

**Figure 4.**
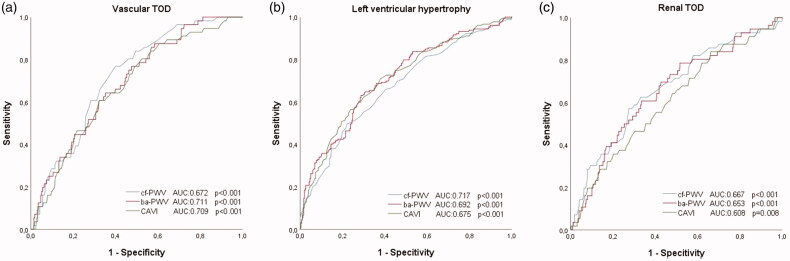
Receiver operating curve of cf-PWV, ba-PWV and CAVI to identify vascular TOD (a), LVH (b) and renal TOD (c). Areas under the ROC curves are summarised in Table 4. TOD: target organ damage; LVH: left ventricular hypertrophy; cf-PWV: carotid-femoral pulse wave velocity; ba-PWV: brachial-ankle pulse wave velocity; CAVI: cardio-ankle vascular index.

Figure 4 and Table 4 show the ROC curves. Thea area under the curve for LVH were: cf-PWV (0.717; CI 95%: 0.656–0.779; *p* < .001), ba-PWV (0.692; CI 95%: 0.625–0.759; *p* < .001) and CAVI (0.675; CI 95%: 0.605–0.745; *p* < .001); for vascular TOD, cf-PWV (0.672; CI 95%: 0.622–0.722; *p* < .001), ba-PWV (0.711; CI 95%: 0.663–0.758; *p* < .001) and CAVI (0.709; CI 95%: 0.661–0.757; *p* < .001); and for renal TOD, cf-PWV (0.667; CI 95%: 0.577–0.729; *p* < .001), ba-PWV (0.653; CI 95%: 0.577–0.729; *p* < .001) and CAVI (0.608; CI 95%: 0.529–0.687; *p* = .008). The cut-off points with the highest sensitivity and specificity for the detection of TOD were, for LVH, cf-PWV (8.25), ba-PWV (13.32) and CAVI (8.35); for vascular TOD, cf-PWV (7.75), ba-PWV (12.78) and CAVI (8.13); and for renal TOD, cf-PWV (8.75), ba-PWV (14.06) and CAVI (8.43).

**Table 4.** Area under receiver operating characteristics curve and cut-off points sensitivity and specificity for the vascular and renal TOD, and LVH.

	Cut-off value	Sensitivity (%)	Specificity (%)	Area under the curve	*p*-value
LVH					
cf-PWV	8.250	0.661	0.673	0.717	<0.001
ba-PWV	13.320	0.643	0.640	0.692	<0.001
CAVI	8.350	0.625	0.633	0.675	<0.001
Vascular TOD					
cf-PWV	7.750	0.615	0.637	0.672	<0.001
ba-PWV	12.775	0.654	0.653	0.711	<0.001
CAVI	8.125	0.659	0.650	0.709	<0.001
Renal TOD					
cf-PWV	8.750	0.500	0.500	0.667	<0.001
ba-PWV	14.062	0.500	0.500	0.653	<0.001
CAVI	8.425	0.500	0.500	0.608	0.008

TOD: target organ damage; LVH: left ventricular hypertrophy; cf-PWV: carotid-femoral pulse wave velocity; ba-PWV: brachial-ankle pulse wave velocity; CAVI: cardio-ankle vascular index.

## Discussion

This study analysed the relationship of arterial stiffness, assessed with three measurements, with the presence of vascular and renal TOD, and LVH in a general population free of cardiovascular disease. The main findings were as follows: a high correlation was found between the different measures of vascular structure and function analysed. In addition, some measures of arterial stiffness was found to be associated with the presence of vascular TOD and LVH. However, this association disappears when cardiovascular risk factors are incorporated as adjustment variables, suggesting that this association is mainly mediated by these variables, especially blood pressure [37].

### Relationship between the different measurements of arterial stiffness

The positive relationship found between the different measurements of arterial stiffness analysed in this work is in line with the results of previous studies conducted with general populations [4,8,11,38] and in individuals with hypertension [23]. However, our results are in disagreement with those published by Alieva et al. [18], which did not show correlations between the different analysed measurements of arterial stiffness. This difference may be due to the type of sample analysed, since the participants of their study were younger, although with higher cf-PWV and lipid profile compared to the individuals of our study.

### Association between the measurements of arterial stiffness and TOD

In the logistic regression analysis of this study, cf-PWV and ba-PWV were positively associated with vascular TOD. These results are supported by the evidence reported in The Northern Shanghai Study of the Chinese population over 65 years of age [19,39] and in patients with hypertension [40]. This is an important finding, since the presence of vascular TOD defined with carotid artery lesion is a useful parameter in predicting future cardiovascular events in hypertensive subjects [41]; nevertheless, it has been little studied in the general population free of cardiovascular disease. A further finding of interest is that there was no association between CAVI and vascular TOD, in contrast to previously reported results in patients with type 2 diabetes mellitus [17].ba-PWV was positively associated with LVH determined with electrocardiographic criteria. These results are in line with those published by Yu et al. [8], which indicate that ba-PWV correlates better with left ventricular mass and diastolic function than cf-PWV, probably because ba-PWV covers a larger arterial tree territory than cf-PWV. Although ba-PWV is considered an index of peripheral arterial stiffness, current evidence supports the role of arterial stiffness in medium-calibre arteries in the development and progression of cardiovascular disease [42].

However, when we add cardiovascular risk factors to the adjustment variables, the association of the three measures of arterial stiffness studied with vascular and renal TOD, and LVH is lost. These results are in line with those in a four-year longitudinal study by Meani et al. [43], which analysed the progression of cf-PWV in hypertensive subjects. In this study, they identified that the two factors responsible for the accelerated progression of cf-PWV after correction for cf-PWV and baseline heart rate were age and blood pressure.

In multiple regression analysis, adjusted for cardiovascular risk factors, cf-PWV was negatively associated with ABI, in line with results published on hypertensive subjects [44]. However, the association with ACR was contrary to what might have been expected. This unexpected association may result from only taking one ACR measure, the variability of this measure, as well as the extreme values potentially influencing these results. Likewise, contrary to what might have been expected, a positive association was observed between ba-PWV and the glomerular filtration rate estimated with the CKD-EPI Equation [45]. These results differ from the study published by Lu et al. [19], in which cf-PWV, but not ba-PWV, were related to glomerular filtration rate. Therefore, the association with the components defining renal TOD are not consistent with previous studies, and these unexpected associations should thus be interpreted with caution.

Finally, the multiple regression yielded a positive association between CAVI and c-IMT. These results are in line with those previously published in subjects with intermediate cardiovascular risk [46], patients with diabetes [17] and hypertension [23].

In sum, the association in the general population free of cardiovascular disease of arterial stiffness measures with vascular and renal TOD, and LVH does not appear to be homogeneous, probably because each of the measures used assesses different pathways of the vascular tree [11]. The underlying mechanisms are not fully established, making further investigation through prospective studies necessary.

Lastly, the findings of this study must be interpreted in the context of its limitations. The transversal character of this analysis does not allow inferring causality, and the population was Spanish, thus it may not be possible to generalise the results to other races or ethnicities. On the other hand, left ventricular hypertrophy has been defined only with electrocardiographic criteria as there are no echocardiographic data.

In conclusion, the results of this study suggest that the different measurements of arterial stiffness analysed are strongly associated with each other. Moreover, we found an association of cf-PWV and ba-PWV with vascular TOD, and of ba-PWV with LVH, although they disappear when adjusting for cardiovascular risk factors.

## Supplementary Material

Supplemental Material

## Abbreviations

cf-PWV: carotid-femoral pulse wave velocity
ba-PWV: brachial-ankle pulse wave velocity
CAVI: cardio-ankle vascular index
TOD: target organ damage
c-IMT: carotid intima-media thickness
LVH: left ventricular hypertrophy
SBP: systolic blood pressure
DBP: diastolic blood pressure
ABI: ankle-brachial index
BMI: body mass index
LDL-c: low-density lipoprotein cholesterol
HDL-c: high-density lipoprotein cholesterol
HbA1c: glycosylated haemoglobin
CKD-EPI: chronic kidney disease epidemiology collaboration
ECG: electrocardiogram
ROC: receiver operating characteristic
AUROC: area under the curve
CI: confidence interval
